# Fibers of Thermoplastic Copolyamides with Carbon Nanotubes for Electromagnetic Shielding Applications

**DOI:** 10.3390/ma14195699

**Published:** 2021-09-30

**Authors:** Paulina Latko-Durałek, Povilas Bertasius, Jan Macutkevic, Juras Banys, Anna Boczkowska

**Affiliations:** 1 Faculty of Materials Science and Engineering, Warsaw University of Technology, 02-507 Warsaw, Poland; paulina.latko@pw.edu.pl (P.L.-D.); anna.boczkowska@pw.edu.pl (A.B.); 2 Technology Partners Foundation, 02-106 Warsaw, Poland; 3 Faculty of Physics, Vilnius University, 10222 Vilnius, Lithuania; pov.bertasius@gmail.com (P.B.); juras.banys@ff.vu.lt (J.B.)

**Keywords:** EMI shielding, dielectric properties, microwaves, carbon nanotubes, hot melt adhesives, polymer-based composites, mechanical properties

## Abstract

Polymer composites containing carbon nanofillers are extensively developed for electromagnetic shielding applications, where lightweight and flexible materials are required. One example of the microwave absorbers can be thermoplastic fibers fabricated from copolyamide hot melt adhesives and 7 wt% of multi-walled carbon nanotubes, as presented in this paper. A broadband dielectric spectroscopy confirmed that the addition of carbon nanotubes significantly increased microwave electrical properties of the thin (diameter about 100 μm) thermoplastic fibers. Moreover, the dielectric properties are improved for the thicker fibers, and they are almost stable at the frequency range 26–40 GHz and not dependent on the temperature. The variances in the dielectric properties of the fibers are associated with the degree of orientation of carbon nanotubes and the presence of bundles, which were examined using a high-resolution scanning microscope. Analyzing the mechanical properties of the nanocomposite fibers, as an effect of the carbon nanotubes addition, an improvement in the stiffness of the fibers was observed, together with a decrease in the fibers’ elongation and tensile strength.

## 1. Introduction

The artificial electromagnetic radiation produced by various electric appliances has become a real problem in the present world. Depending on the type of device, electromagnetic waves occur in a broad frequency range, from 10^4^ to 10^12^ Hz. Unfortunately, more and more devices work in the same frequency range, resulting in waves accumulating in the form of so-called electromagnetic pollution or electromagnetic interference (EMI). Especially harmful is high power microwave radiation, which occurs in the range between 30 MHz and 300 GHz and is emitted by a significant number of devices such as mobile phones, microwave ovens, televisions, satellite communications, Wi-Fi, FM radio broadcasts, Bluetooth, wireless local area network (LAN), radars, global positioning system (GPS), or marine and military systems. Interaction and interference of the microwaves cause devices to malfunction and have an impact on the human central nervous system. Therefore, seeking and developing shielding materials that show high effectiveness has become increasingly examined in the last decades, and the research is fully justified [1,2,3,4,5,6,7].

The standard materials used to shield a broad spectrum of electromagnetic waves are metals (copper, silver, nickel, gold) and products made from them, such as tapes or meshes. Due to their high electrical conductivity (>10^6^ S/m), metals can shield electromagnetic pollution, but they have some disadvantages from a technical point of view. They are heavy, difficult to process, corrosive, and not flexible, limiting the formation of various shapes, and their properties are not easily modified. Therefore, polymer-based composites are being extensively developed as alternative shield materials. Due to the presence of thermoplastic polymer as a matrix, they can be processed into various shapes using commercial techniques (e.g., extrusion, injection molding). They possess a high degree of flexibility, high chemical and corrosion resistance, and low weight associated with the polymers’ low density. In contrast with metals, the morphology of polymer composites can be easily tailored by changing the type of filler, its concentration, the polymer’s properties (e.g., viscosity, crystallinity), and finally, by processing conditions. Moreover, unlike metals, in which EMI shielding is based on the reflection phenomenon, polymeric materials demonstrate the ability to shield electromagnetic waves primarily through absorption, which is preferred, for instance in military or stealth technology [1].

Various types of conductive fillers and nanofillers are used to transform the polymers, which are insulators, into electrically conductive materials. It has been shown that the most promising materials for EMI shielding applications should possess high electrical conductivity and excellent dielectric and magnetic properties. Such multifunctionality allows for the electromagnetic waves to be shielded effectively by reflection and adsorption mechanisms. In addition, the possibility of offering porous materials (e.g., conducting composites with fillers, foams, or honeycombs) allows EMI shielding by the multiple reflections on different surfaces to be achieved [3,8,9,10]. The commonly used fillers are metals and metal oxides in the form of powders or particles (e.g., Ni, Cu, Fe, Co, ZnO, SiO_2_, TiO_2_, BaTiO_3_, etc.), which are mixed with the polymers or are applied in the form of coatings [7,10,11]. The other possibility is to use fillers for the polymers in the form of short fibers such as carbon fibers, metal-coated carbon fibers, or stainless steel fibers [12,13]. However, to obtain high electrical or dielectric properties, they need to be added at high concentrations, which causes limitations in their processing by standard industrial techniques and increases the weight of the composite. From the newest solutions, electrically conductive polymers such as polyaniline and 2-D inorganic layered materials, called M-Xenes, have been tested with respect to their EMI shielding ability [14,15].

Much more attention is currently being focused on carbonaceous fillers and nanofillers, which, due to their high electrical conductivity, can improve the dielectric properties of polymers. The materials within this group that have been studied extensively are single-walled carbon nanotubes, multi-walled carbon nanotubes, carbon nanowires, graphite nanoplates, graphene oxide, reduced graphene oxide, graphene nano-sheets, graphene foam, and aerogels [14,16]. Since they have a high aspect ratio, their percolative network is achieved at low concentrations, resulting in an electromagnetic shielding ability being reported for many various polymers. In addition, a small amount of carbon nanofillers does not increase the total weight of the composite. Finally, the polymers enhanced by carbon nanofillers exhibit superior mechanical and thermal properties and a wide range of multifunctionalities. The main difficulty is that carbon nanofillers occur as agglomerates, which need to be destroyed during the process of mixing with polymers. Only nanofillers well-dispersed in the polymer matrix result in the formation of a percolation network able to conduct electric current and shield electromagnetic waves. Due to the modification of the processing parameters, the state of nanofillers’ dispersion can be controlled, and the final composite properties have a tunable morphology [17,18]. This, together with the chemical and corrosion resistance, low weight, flexibility, ease of processing into various shapes, and inexpensiveness, is why polymer composites have the potential to replace the metallic materials in EMI shielding applications [2,17,19,20,21,22,23,24]. It should be noted that the development process of innovative EMI shielding materials is also focused on their final form—ready-to-use in industrial applications. A good microwave absorber should be thin and lightweight. The most common forms are foils, films or papers [21,22], coatings [25,26,27], fibers [23] or textile products such as woven or nonwoven fabrics or mats [24,25]. They were applied in fiber-reinforced composites as an alternative to metallic meshes or tapes [17].

This paper describes a novel type of nanocomposite fibers manufactured from three kinds of copolyamide hot melt adhesives (HMAs) and multi-walled carbon nanotubes (MWCNTs) by a melt-spinning process. The main goal of the work was to analyze the dielectric properties of the single nanocomposite fiber in correlation with the addition of MWCNTs and the type of polymer matrix. Moreover, the dielectric properties were measured for fibers with various diameters at the gigahertz range and variable temperatures. According to our knowledge, no papers describe the dielectric properties of fibers consisting of thermoplastic polymers and carbon nanofillers. There is some work related to the measurement of the dielectric properties of single carbon fibers [26] and lignocellulose fibers coated with polyaniline [27] and metal oxide [28]. Understanding the relationship between the morphology of the copolyamide nanocomposite fibers and measured dielectric properties will help select those with the highest ability to shield electromagnetic waves. The possible application of these fibers is to use them as a precursor in the fabrication process of woven or nonwoven fabrics by stitching, pressing, or spun-bond processes.

## 2. Materials and Methods

Three types of copolyamide hot melt adhesives (coPA HMAs) supplied in pellet form by EMS Griltech (Domat, Switzerland) were used as the polymer matrix. They contained a randomly arranged segment of polyamide 6 and polyamide 6; however, they differed in melt viscosity and melting range (Table 1). MWCNTs (trade name: NC7000, Nanocyl, Sambreville, Belgium), having a diameter of 9.5 nm and a length of 1.5 µm (aspect ratio–158), were used as the electrically conductive nanofiller. They were synthesized via a catalytic carbon vapor deposition process, with a purity of 90%. Their surface area was 250–300 m^2^/g, and electrical volume resistivity was 10^−4^ Ω⋅cm. All copolyamides were mixed with a powder of MWCNTs in a concentration of 7 wt% of MWCNT using a twin co-rotating extruder at 200 °C, with screw rotation of 200 rpm. The concentration 7 wt% of MWCNT was used because it resulted in high DC electrical conductivity of composites, as shown previously [29]. The nanocomposite pellets (coPA + MWCNTs) were delivered by Nanocyl.

**Figure 1:**
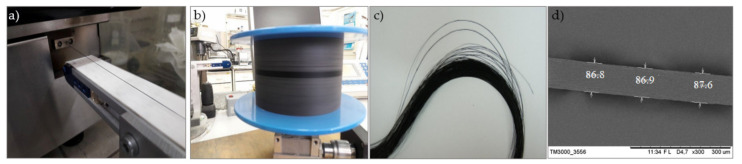
The equipment used to produce nanocomposite fibers: (**a**) extruder with a forming fiber; (**b**) fibers on a winding reel; (**c**) produced nanocomposite fibers; (**d**) an example fiber with measured diameters.

Nanocomposite fibers were produced directly from pellets of copolyamides containing 7 wt% MWCNTs, while neat fibers were manufactured directly from copolyamide pellets. For the preparation of the samples, a laboratory twin-screw extruder HAAKE MiniLab (ThermoFisher Scientific, Waltham, MA, USA) was used. The minimum amount of material loaded into the barrel was 5 g. The extruder was equipped with two conical screws with a length of 109.5 mm, width of 14 mm, and 5 mm at its narrowest point. The melt-spinning process of the fibers was performed using a round shape nozzle of 0.75 mm connected to the extruder. The pellets, after loading, were melted and then extruded through the nozzle (Figure 1a). After the extruder, the fibers were placed on a transport belt and then wound on a reel rotating at high velocity (Figure 1b). Table 2 presents the melt-spinning process parameters applied. It was found that the optimum screw velocity was 20 rpm for neat fibers and 30 rpm for fibers with MWCNTs; in turn, the speed of the reel on which the fibers were collected was 200 rpm for neat fibers and 300 rpm for fibers containing MWCNTs. Obviously, screw velocity and winding reel speed both affect the diameter of the fibers. By applying a high-velocity winding reel, the fibers could have diameters as low as 50 μm, but they often broke during manufacturing. Therefore, both velocities were optimized to produce fibers continuously (Figure 1c). Applying these parameters allowed neat fibers with diameters smaller than for the fibers containing MWCNTs to be obtained. The average fiber diameters were calculated from scanning microscope images, and they are presented in Table 2, while an example is presented in Figure 1d. In order to analyze the effect of the fibers on the dielectric properties, nanocomposite fibers were produced with a variable screw velocity (100, 70, 40, and 20 rpm). This made it possible to obtain fibers with different diameters (from 90 to 200 µm).

The diameters of the produced nanocomposite fibers were characterized using a scanning electron microscope (SEM 3000 Hitachi High-Tech, Tokio, Japan) with the applied voltage of 5 kV. Fibers manufactured from neat copolyamides were coated with a thin layer of gold. To obtain the average value, at least 40 fibers were measured, and the average diameter was calculated (Table 2). The dispersion and arrangement of MWCNTs in the nanocomposite fibers was examined applying a high-resolution transmission electron microscopy (HR STEM S5500, Hitachi High-Tech, Tokio, Japan). Single fibers were embedded in epoxy resin, and after curing, they were cut into slides with a thickness of approximately 80 nm using an ultramicrotome (EM UC6, Leica Microsysteme, Vienna, Austria) equipped with a diamond knives. The slides were cut at −100 °C with a cutting speed set at 1 mm/s. The plane of the cross sections was aligned with the direction of the extrusion process, and the surface of the fibers was also analyzed. The observations were performed at a voltage of 30 kV.

Microwave dielectric spectroscopy was used to characterize the dielectric properties of the produced fibers according to the method described for thin cylindrical rods [24]. The moduli of microwave reflection and transmission coefficients were measured with an automatic dielectric spectrometer in the frequency range from 8 to 37 GHz. Fibers with a cylindrical shape were placed in the center of the wide waveguide wall, parallel to the electrical field of the main TE_10_ modes. A special sample holder containing a slot for pistons was used. A notch was made in the piston for the contacts between the sample and the waveguide. Two other pistons were used for calibration of reflections and transmissions. This method prevented destruction of the waveguide channel to be rejected and made it possible to verify the calibration during the experiment. The complex dielectric permittivity of samples was calculated according to the method presented in [24]. For measurements at higher temperatures a home-made furnace was used, while for measurements at low temperatures liquid nitrogen was used.

**Figure 2:**
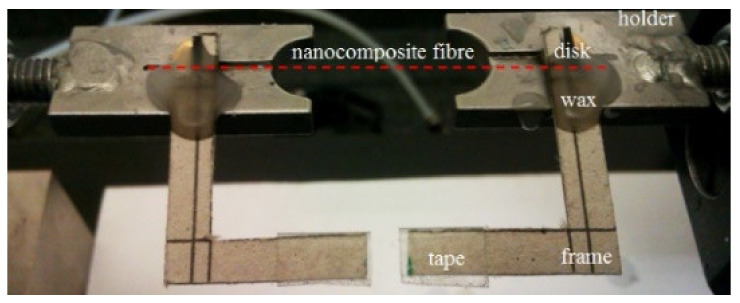
Frame with fixed fibers on the machine’s holder.

The mechanical properties of the fibers were measured in tensile testing (MTS Tytron 250 Instrument) according Standard Test Method for Tensile Strength and Young’s Modulus of Fibres (ASTM C1557-03 (2013)). First, nanocomposite fibers were fixed to a special thick paper frame equipped with metal disks. Then, the frame was clamped to the machine’s holder using epoxy glue, and after solidification, the frame was cut directly before the test (Figure 2).

From each type of material, 15 single fibers were tested to failure at a constant cross-head displacement rate at a velocity of 10 mm/min. The values of tensile strength (*R_m_*), elongation at break, and Young’s modulus (*E*) were then calculated from the formulas: $R_{m}=\frac{F_{m}}{A_{0}}\;\left(\mathrm{MPa}\right)$ and $E=\frac{F\times l_{0}}{A_{0}\times \Delta l\;}\;\left(\mathrm{MPa}\right)$, where *F_m_* is maximal force (N), *A*_0_ is the diameter of the fiber (mm), *F* is force determined from linear region of *F* (Δ*l*) curve (N), *l*_0_ is the length of a base (35 mm), and Δ*l* is elongation at break (mm). Results were calculated taking into account the value of fiber diameter.

## 3. Results

### 3.1. Microstructure

The fibers from neat copolyamides and from their nanocomposites containing 7 wt% MWCNTs were successfully produced by a melt-spinning process. Fabrication of neat fibers was easier than fabrication of those with 7 wt% MWCNTs, resulting in lower diameter and lower standard deviation in the case of unfilled fibers (see Table 2). In the presence of 7 wt% MWCNTs, the production of fibers was slightly more difficult because they broke much more often, probably due to presence of local MWCNTs agglomerates or bundles, which decreased their mechanical strength. The most problematic in the melt-spinning process was coPA_B + 7 wt% MWCNTs, and therefore the produced fibers had the highest diameter (approximately 160 µm, Table 2). In contrast, coPA_A + 7 wt% MWCNTs and coPA_C + 7 wt% MWCNTs were both easy to process into the form of fibers with diameters of around 100 µm. Such discrepancies in fiber diameter are related to the state of dispersion of each copolyamide in the starting nanocomposite pellets of. In our previous paper, we observed that the biggest agglomerates occurred in coPA_B + 7 wt% MWCNT (90 µm) and their content was around 11% [29]. The presence of big agglomerates made it necessary to apply a lower drawing ratio due to the tendency of the fibers to break. In the case of coPA_A + 7 wt% MWCNTs nanocomposite pellets, agglomerates had rather small diameters of around 30 μm, so they did not hamper the extrusion process so much. This resulted not only in the lowest average diameters but also in a much smoother surface of coPA_A + 7 wt% MWCNTs fibers examined by SEM, which is shown in Table 3. The presented images also show that neat fibers had much smoother surfaces than fibers containing 7 wt% MWCNTs. Nanocomposite fibers of copolyamides all had uneven surfaces. These defects were caused by the presence of MWCNT agglomerates in the initial nanocomposite pellets, which changed the flow of the material from the extruder nozzle, thus creating a rough surface and deviations in the diameters of fibers.

**Figure 3:**
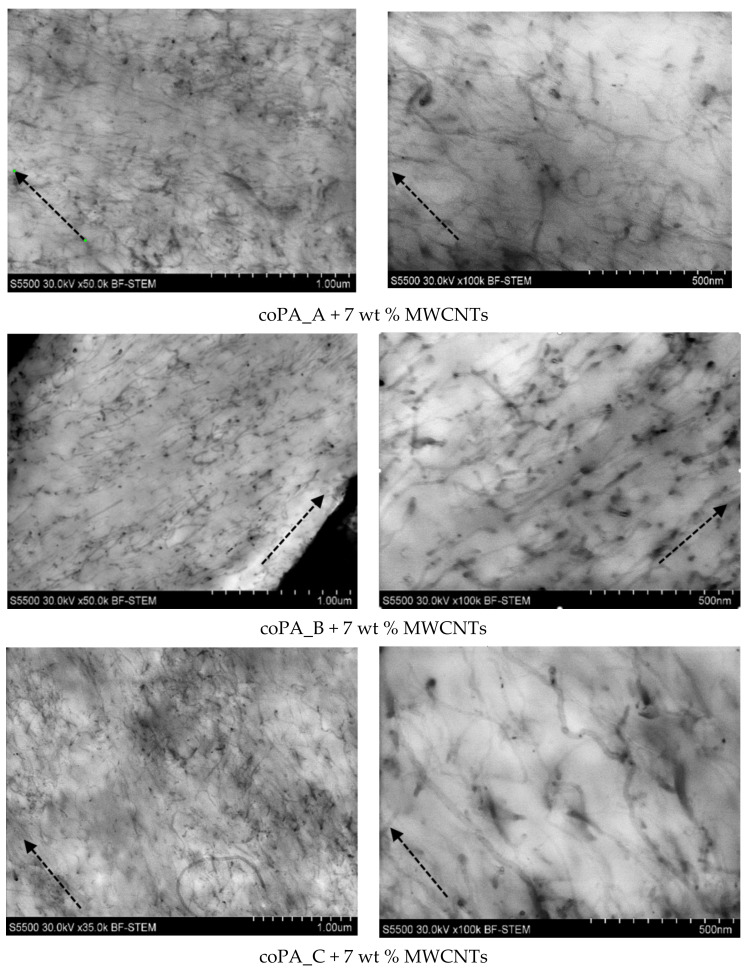
The state of MWCNTs’ dispersion and their arrangement in the nanocomposite fibers. The images were captured using a high-resolution microscope, along the axis of the fiber.

It is known that during extrusion and drawing, carbon nanotubes (CNTs) can be oriented along the direction of the fibers to various degrees. The orientation of the CNTs during extrusion is caused by the material shear direction, which results in anisotropic properties of the fibers [30,31]. The dispersion and orientation of MWCNTs in the produced nanocomposite fibers were analyzed using images taken from a high-resolution microscope (Figure 3). It can be seen that MWCNTs were indeed oriented in the direction of the axis of the fibers (marked with arrows); however, the level of alignment seemed to be the highest for coPA_B + 7 wt% MWCNTs. Long single tubes could be recognized in all fibers; nevertheless, some of the MWCNTs occurred in an entangled state. Especially in the fibers of coPA_A + 7 wt% MWCNTs and coPA_C + 7 wt% MWCNTs, there were visible bundles or clusters with loosely connected tubes. Low alignment of the MWCNTs in the fibers’ axis indicates that the applied winding reel velocity was insufficient. However, increasing the reel speed posed a problem due to the brittleness of the fibers caused by the high content of MWCNTs. Nevertheless, the direct manufacturing of fibers from the masterbatches is a promising method for application in industrial conditions.

### 3.2. Dielectric Properties

The basic properties possessed by materials that are promising for EMI shielding are the ability to conduct electric current and high dielectric permittivity. In order to analyze these properties in the manufactured nanocomposite fibers, a highly sensitive broadband dielectric spectroscopy was applied. Generally, addition of various organic and inorganic fillers improves the intrinsic dielectric properties of polymers, as described in the literature for many various polymers [32]. For the nanocomposites based on copolyamides, fabricated in the form of pellets, it was already shown that MWCNT additions had a positive effect on the improvement of AC electrical conductivity and permittivity. It was found that 7 wt% of MWCNTs resulted in percolated structure in HMAs and therefore, conductivity properties [33]. In this paper, the dielectric properties were measured for single fibers manufactured from pellets of these nanocomposites.

**Figure 4:**
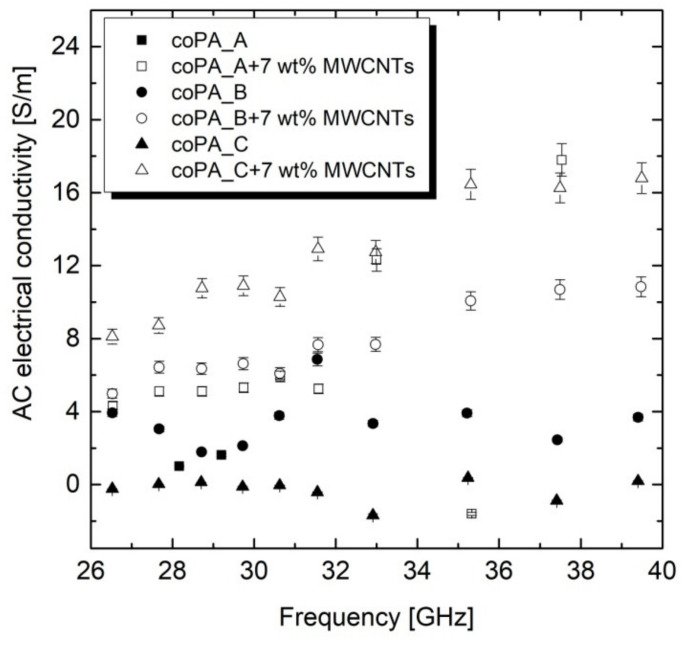
AC electrical conductivity measured in GHz frequency for the neat fibers and those containing 7 wt% MWCNTs. The fibers for the analysis had a similar diameter of around 100 µm.

The dependence of AC electrical conductivity for the neat fibers and those doped with 7 wt% MWCNTs is presented in Figure 4. It was revealed that the addition of 7 wt% of MWCNT to the copolyamides increased the AC electrical conductivity in the fibers produced by the melt-spinning process in the entire investigated frequency range from 26 to 40 GHz. However, there was a strong effect of the frequency on the conductivity property in the fibers. At lower frequency (below 32 GHz), the value of the conductivity was between 0 and 4 S/m for neat fibers, and between 4 and 8 GHz for the fibers containing 7 wt% MWCNTs. At higher frequencies (above 32 GHz); there was an observable increase in the AC conductivity up to 17 S/m for the nanocomposite fibers of coPA_A + 7 wt% MWCNTs and coPA_C + 7 wt% MWCNTs, and up to 10 S/m for coPA_B + 7 wt% MWCNTs fibers. Such a difference can be related to the electron tunneling conductivity in nanocomposites [3], which is different for pure matrices, where electrical properties are mainly related to polymer relaxation [32], and the MWCNT arrangement and orientation in the nanocomposite fibers, as shown in Figure 3. Based on the microstructure observations, it can be stated that the visible bundles of MWCNT observed in coPA_A and coPA_C fibers positively affected the AC conductance. This is unlike coPA_B + 7 wt% MWCNTs fiber, where MWCNTs occur in a well-dispersed state and recognized orientation along the fibers’ axis. As described in the literature, the electrical conductivity in thermoplastic polymer/CNT fibers can be lost due to the lack of a CNT–CNT network [30]. On one hand, applying an additional stretching step allows the bundles of CNTs to be to exfoliated and oriented, thus leading to highly conductive fibers. On the other hand, too high a draw ratio can destroy the CNT–CNT network and reduce the electrical conductivity [34].

**Figure 5:**
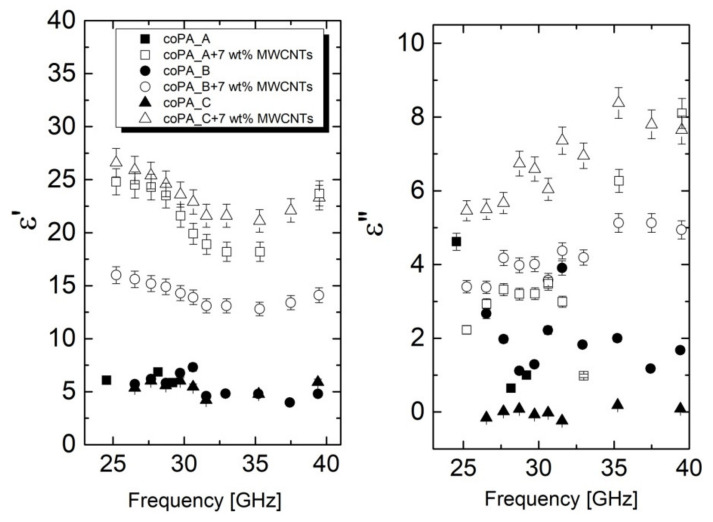
Frequency dependence of complex dielectric permittivity for copolyamide-based fibers with and without MWCNTs. The fibers for the analysis had a similar diameter of around 100 µm.

Microwave complex dielectric properties of materials are very important for EMI shielding applications. The most suitable materials should exhibit optimal microwave dielectric properties [15]. As shown in Figure 5, the dielectric permittivity was between 4 and 7 for neat fibers, which is a typical low value of dielectric permittivity for various polymers, including polyamides [35]. Moreover, the obtained measuring points for the neat fibers were close to each other, revealing the negligible effect of the matrix composition on the dielectric properties. Significant changes in the real permittivity were observed for the fibers containing MWCNTs. For them, the dielectric permittivity increased up to 24 for the fibers based on coPA_A and coPA_C. In the case of coPA_B + 7 wt% MWCNTs, the dielectric permittivity was lower—around 16. These results confirm the correlation between the electrical conductivity and the dielectric permittivity in the studied fibers. The lower the conductivity, the lower the permittivity, as shown for the composites based on coPA_B. Therefore, the changes in the dielectric properties of the nanocomposite fibers were dependent mainly on the state of dispersion, orientation, and arrangement of the nanofiller. The minimum of dielectric permittivity close to 34 GHz (Figure 5) obviously was related to some resonance effect in the waveguide system [9].

**Figure 6:**
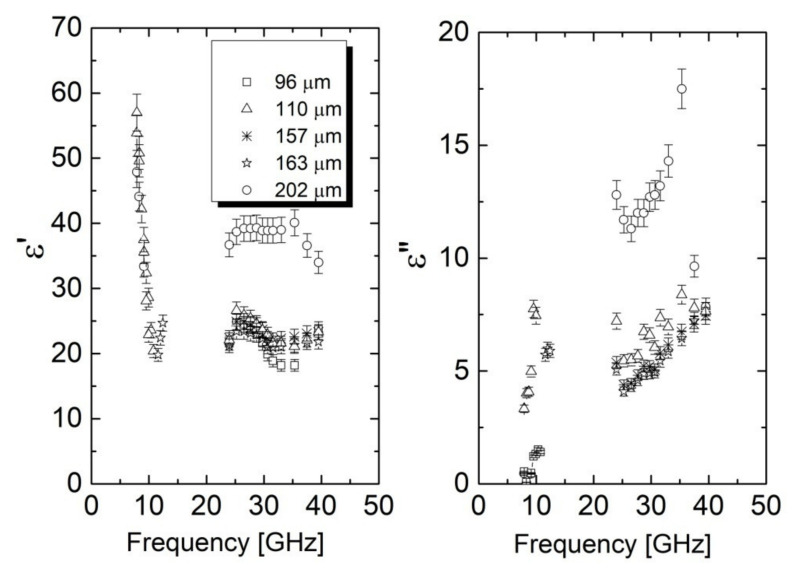
Frequency dependence of complex dielectric permittivity for coPA_A + 7 wt% MWCNTs fibers with different diameter.

**Figure 7:**
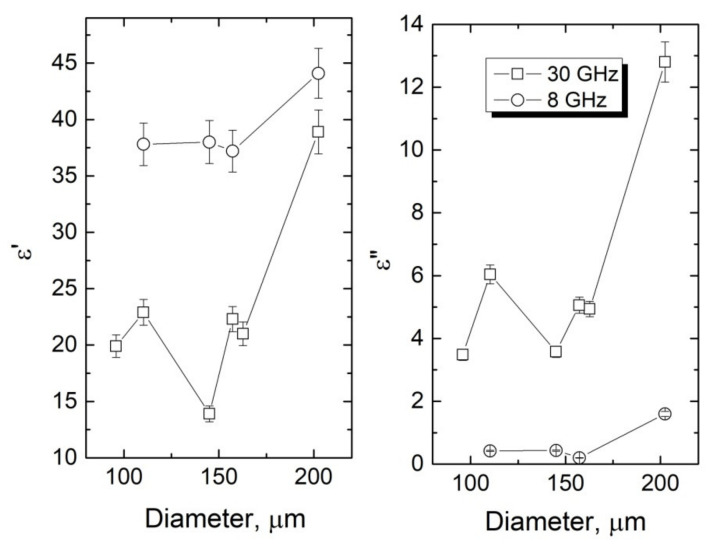
Complex dielectric permittivity versus coPA_A + 7 wt% MWCNTs fibers diameter.

Since the highest electrical conductivity was achieved for coPA_A + 7 wt% MWCNTs, that material was selected to fabricate fibers with various diameters. The purpose was to analyze the effect of the diameters of the fibers on the dielectric properties. The microwave spectra of complex dielectric permittivity results for coPA_A + 7 wt% MWCNTs fibers with different diameters are presented in Figure 6. It can be seen that the value of complex dielectric permittivity was strictly dependent on the diameter of the fiber. In the fibers with the lower diameters (96, 110, 157, and 162 µm) the dielectric permittivities were close to each other (for example, ε′ = 18.2–22.6 and ε″ = 6.27–8.4 at 35 GHz). A significant jump was observed for the fibers with a diameter of 202 µm at all investigated frequencies (Figure 6 and Figure 7). At 30 GHz, the dielectric permittivity was ε′ = 40 and ε″ = 13, which is a relatively high value (Figure 7). Such an effect is associated with the MWCNTs content and their dispersion. In the thicker fiber, there were more MWCNTs per unit of volume than in the thin fiber, and they were less oriented [36]. Owing to this, more local fields where MWCNTs polarized with a polymer matrix were formed, thus increasing the permittivity of the nanocomposite fibers [26]. For plate-like samples with such dielectric properties and thickness of 1 mm, huge electromagnetic shielding of about 50% was observed [37]. Therefore, the plate-like shield produced from the fibers under investigation should have exhibited optimal electromagnetic shielding. Moreover, the complex dielectric permittivity was almost frequency independent in the 26–40 GHz frequency range and was easily controlled by fiber diameter. Therefore, broadband 3D microwave absorbers can be designed experimentally and theoretically [38]. One of the possibilities is to produce nonwovens directly from such fibers and to place them into the fiber-reinforced polymers that have been presented in our previous papers [39,40].

**Figure 8:**
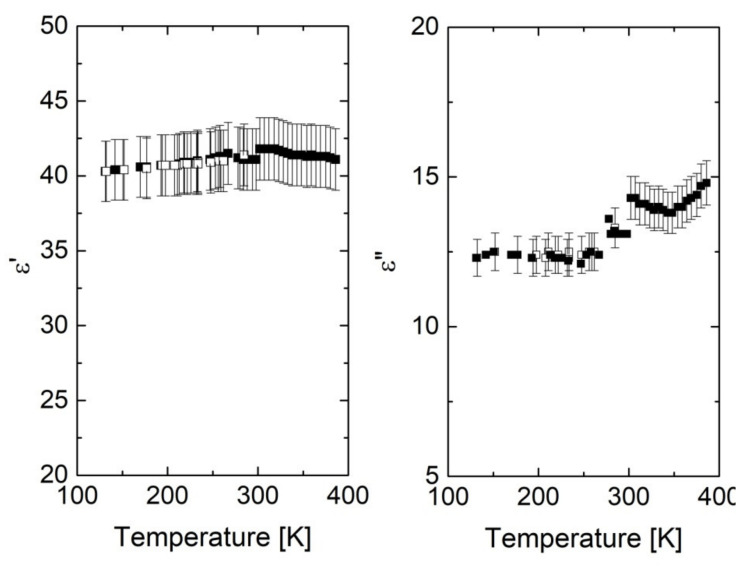
Temperature dependence of the complex dielectric permittivity for the nanocomposite fiber made of coPA_A + 7 wt% MWCNTs having diameter of 202 µm (solid symbols results of measurements on heating, open on cooling). The measurement frequency was 30 GHz.

The dielectric properties of the nanocomposite fibers made from coPA_A + 7 wt% MWCNTs with diameter 202 μm were investigated in a wide temperature range (120–400 K). The results are collected in Figure 8. It can be seen that dielectric properties were almost temperature-independent in a wide temperature range. A slight increase in dielectric losses ε″ above room temperature was obviously related to premelting effect (liquid materials are usually more electrically conductive), while the maximum of dielectric permittivity was connected with Maxwell–Wagner relaxation [41]. The weak scattering in the temperature dependence of dielectric properties is related to measurement accuracy, which was not worse than 5%. It should be noted that no hysteresis was observed in the data on heating and cooling, indicating that structures were quite stable in the 120–400 K temperature range.

### 3.3. Mechanical Properties

**Figure 9:**
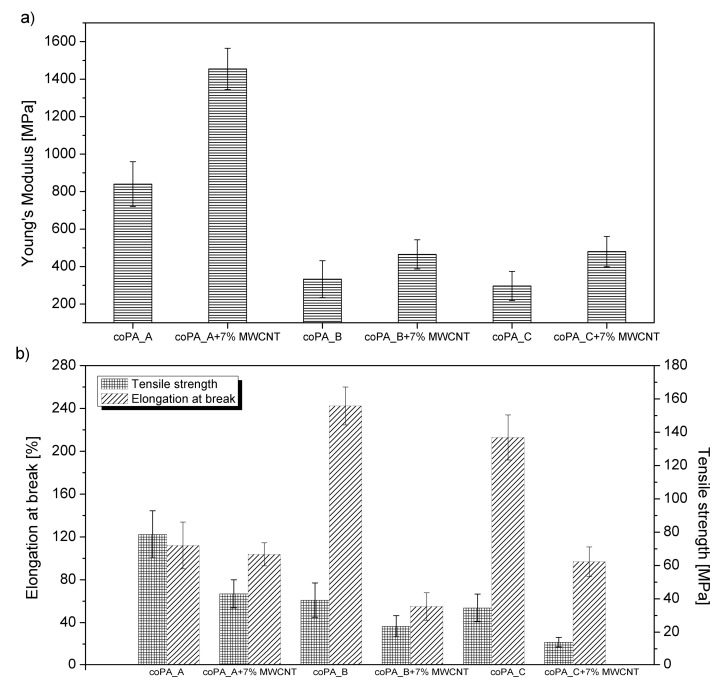
Comparison of mechanical properties of produced fibers (**a**) Young’s modulus; (**b**) tensile strength, and elongation at break.

It has been observed that the mechanical properties of many polymeric fibers containing CNTs are enhanced, but the degree of improvement is dependent on CNT dispersion, alignment, or the type of polymer used. Therefore, the produced neat fibers and those containing 7 wt% MWCNT were characterized with respect to their mechanical properties. The results are presented in the form of graphs in Figure 9. Figure 9a shows that the addition of 7 wt% MWCNTs improved Young’s modulus for each type of copolyamide. The highest increase in Young’s modulus from 840 to 1455 MPa was achieved for coPA_A, after the addition of 7 wt% MWCNTs. Much lower values of Young’s modulus were achieved for coPA_B + 7 wt% MWCNTs (465 MPa) and coPA_C + 7 wt% MWCNTs (480 MPa). According to the literature, such values are rather low since for many thermoplastic fibers filled with MWCNTs the Young’s modulus is measured in GPa. For instance, fibers of PA6 with 1 wt% of MWCNTs produced by single screw extrusion had a Young’s modulus of 3.6 GPa [37]. The authors reported that such excellent stiffness of the fibers was achieved due to the perfect alignment of the MWCNTs along the axis of the fibers. The same fibers also showed improvement in tensile strength from 70 to 115 MPa. In the case of copolyamide fibers in the presence of 7 wt% MWCNTs, the tensile strength was lower than for neat fibers (Figure 9b) and was the highest for coPA_A + 7 wt% MWCNTs. Similarly, all nanocomposite fibers of copolyamides containing 7 wt% MWCNTs had decreased elongation at break. However, for coPA_A, this decrease was only about 20%, which is consistent with the highest Young’ modulus being observed for coPA_A + 7 wt% MWCNTs. Decreasing the elongation at break for coPA_B and coPA_C was much more significant, about 180%, coming from the lower flexibility of the polymer chains in the presence of high content of MWCNTs [42]. According to the literature, the mechanical properties are improved for most fibers containing CNTs—however, only when the concentration of MWCNTs is low. One such example is fibers of poly (lactic acid), for which the addition of MWCNTs does indeed increase the tensile strength, but only up to 3 wt% of MWCNTs [43]. The higher content of MWCNTs in the fibers means there is a high likelihood of the formation of agglomerates or bundles, which act as stress centers that decrease the mechanical properties. Additionally, the low mechanical strength of the presented nanocomposite fibers can also be connected with insufficient alignment and orientation of MWCNTs in the fibers. It can be associated with a too high viscosity of the matrix and insufficient draw ratio applied during the manufacturing process. Nevertheless, the results of mechanical test revealed that coPA_A had the strongest interfacial bonding, which was responsible for improving the mechanical performance of composites with CNTs [41].

Beyond the microscopic effects on the mechanical properties of the fibers, there were also macroscopic effects related to the quality of the surface of the fibers. As shown in Table 3, neat fibers had much smoother surfaces than fibers containing 7 wt% MWCNTs. Fibers of coPA_A + 7 wt% MWCNTs, coPA_B + 7 wt% MWCNTs, and coPA_C + 7 wt% MWCNTs all had uneven surfaces caused by MWCNT agglomerates. Therefore, the tensile strength of the nanocomposite fibers was reduced because the surface defects acted as notches that caused the fibers to rupture much more quickly. This strong relation between the surface defects and the mechanical properties was also found in fibers of polyvinyl alcohol/SWCNT nanofibers [44] and poly (methyl methacrylate) electrospun with 5 wt% MWCNTs [45]. It was revealed that the density of surface defects affected the strength of the fibers, but only for those with diameters above 4 μm. Additionally, the surface defects did not affect Young’s modulus; this can be explained by the increased stiffness of coPA + 7 wt% MWCNT fibers, with a simultaneous reduction in elongation at break and tensile strength.

## 4. Conclusions

Thermoplastic nanocomposite fibers with MWCNTs were successfully developed with outstanding electromagnetic properties. These fibers were fabricated from hot melt adhesives and 7 wt% of MWCNTs by the melt-spinning process. Analysis of their dielectric properties in the gigahertz range showed that the fibers possessed a weak dielectric permittivity dependence in the frequency range of 26–40 GHz; therefore, they can be potentially applied in microwave electronic devices. The achieved dielectric permittivity and electrical conductivity values in microwave frequency range for the fabricated fibers were high enough (ε′ up to 24 and AC electrical conductivity up to 17 S/m) for the structures made of these fibers—for example, thin fabrics—to exhibit EMI shielding performance. Moreover, it was shown that the dielectric permittivity increased together with the diameter of the fiber, and it was almost independent of the temperature variable. Using a high-resolution microscope, it was found that MWCNTs were aligned along the fiber axis; however, the degree of alignment was not the same for each type of fiber. Because of that, the measured AC electrical conductivity was the highest (17 S/m) for the fibers based on coPA_A and coPA_C where MWCNTs were not perfectly oriented but when they remained as bundles. This affected the value of the dielectric permittivity, which was the highest for coPA_A- and coPA_C-based fibers. The mechanical analysis results show that fibers based on coPA_A had higher mechanical strength than those fabricated from coPA_B and coPA_C. What is more, in the presence of 7 wt% MWCNTs, the Young’s modulus increased for all nanocomposite fibers, but primarily for fibers manufactured from coPA_A (up to 1400 MPa). The tensile strength and elongation at break was lower for the nanocomposite fibers in comparison with the neat ones.

## Figures and Tables

**Table 1:** Properties of copolyamides used as a polymer matrix.

Trade Name	Designation	Melting Range (°C)	Melt Viscosity (Pa⋅s)	Melt Volume Rate (cm^3^/10 min) 160 °C/2.16 kg
Griltex^®^1330A	coPA_A	125–135	1200	9
Griltex^®^2A	coPA_B	120–130	600	18
Griltex^®^1566	coPA_C	115–125	150	70

**Table 2:** The melt-spinning conditions to produce nanocomposite fibers.

Nanocomposite Fibers	Extrusion Temperature (°C)	Screw Velocity (rpm)	Winding Reel Velocity (rpm)	Average Diameter (µm)
coPA_A	160	20	300	92.6 ± 9.5
coPA_A + 7 wt% MWCNTs	195	30	300	98.7 ± 19.1
coPA_B	145	20	300	71.5 ± 5.3
coPA_B + 7 wt% MWCNTs	195	30	260	157 ± 26.7
coPA_C	160	20	305	96.3 ± 8.5
coPA_C + 7 wt% MWCNTs	200	30	305	110 ± 14.9

**Table 3:** The comparison of the surface of fibers fabricated from neat copolyamides and from copolyamides containing 7 wt% MWCNTs.

Polymer Matrix	Neat Fiber	With 7 wt% MWCNTs
coPA_A	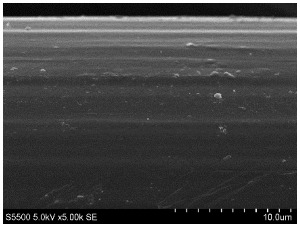	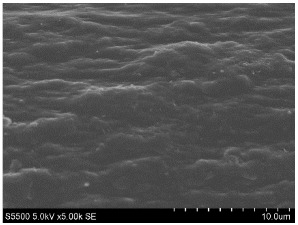
coPA_B	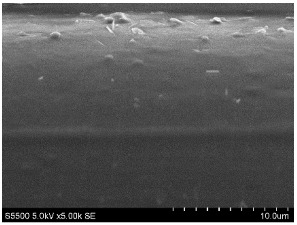	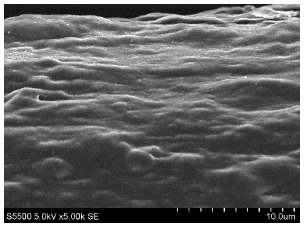
coPA_C	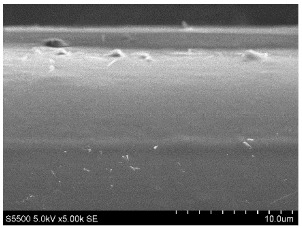	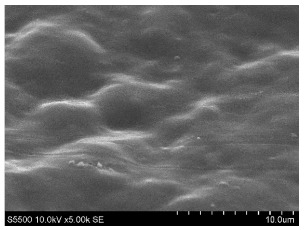

## Data Availability

The data presented in this study are available on request from the corresponding author.
